# Post-traumatic recurrent ulnar nerve dislocation at the elbow: a rare case report

**DOI:** 10.1097/MS9.0000000000001651

**Published:** 2023-12-19

**Authors:** Manh Nguyen Huu, Quyet Tran, Viet Vu Duc, Dung Tran Trung

**Affiliations:** a Department of Orthopaedic Surgery, Vin University; b Orthopaedic and Sports Medicine Center, Vinmec Hospital, Hanoi, Vietnam

**Keywords:** Cubital tunnel syndrome, elbow injury, medial epicondylitis, ulnar nerve dislocation, ulnar nerve transposition surgery

## Abstract

**Introduction and importance:**

Several authors have also made reference to a less prevalent condition known in elbow as ulnar nerve subluxation. However, this particular condition tends to manifest primarily in young individuals who engage in professional sports or activities involving extensive use of the forearm. A more severe form of ulnar nerve subluxation, which is ulnar nerve dislocation, gives rise to a characteristic dislocation and relocation of the nerve at the elbow during flexion and extension of the forearm. Due to the rarity of this condition in clinical settings and its predominant occurrence as subluxation in younger patients, there are instances where traumatic ulnar nerve dislocation can be overlooked and misdiagnosed with two commonly encountered pathological conditions as ulnar nerve entrapment or medial epicondylitis.

**Case presentation:**

The authors present a 51-year-old male with chronic pain when moving his right forearm following a fall that caused a direct force injury to his elbow. The patient was misdiagnosed and treated as medial epicondylitis and early-stage ulnar nerve entrapment. However, the symptoms did not improve for a long time. The authors performed the ulnar nerve anterior transposition surgery using the subcutaneous transposition technique and the result is very good without any pain.

**Clinical discussion:**

The ulnar nerve can naturally be subluxed or dislocated if Osborne’s ligament is loose or when there are anatomical variations in the medial epicondyle. In some case, this ligament can be ruptured by trauma. The symptoms of ulnar instability are caused by friction neuritis. Dynamic ultrasound of the ulnar nerve in two positions show clearly this condition.

**Conclusion:**

Post-traumatic ulnar nerve dislocation is a rare condition, and the recurrent characteristic of it leads to neuritis or neuropathy. The condition can be overlooked or misdiagnosed as medial epicondylitis or early-stage ulnar nerve entrapment. The nerve transposition surgery will give good result.

## Introduction

Highlights

- Ulnar dislocation occurs naturally or after trauma. The patient’s typical symptom is irritation of the ulnar nerve due to friction.
- Ulnar dislocation can be misdiagnosed by ulnar nerve entrapment or medial epicondylitis.
- Dynamic ultrasound a simple subclinical and avoids missing this lesions.

Medial epicondylitis is one of the most common pathologies at the elbow, second only to lateral epicondylitis, accounting for ~10% of all epicondylitis cases^1^. Another commonly encountered condition in the cubital region is ulnar nerve entrapment, which is the second most prevalent peripheral nerve disorder of the elbow after median nerve entrapment. Based on current literature, medial epicondylitis and ulnar nerve entrapment are the two most frequently considered pathologies for chronic pain at the cubital region, with varying alterations of sensation and motor functions of the forearm, wrist, and hand depending on the specific condition.

Contrary to the prevalence of the above two pathologies, ulnar nerve dislocation in the cubital region is relatively rare and can be caused by several factors, such as natural laxity, anatomical variations of the medial epicondyle and retrocondylar groove, and congenital disorders^2^. This condition is commonly seen in young athletes involved in sports requiring significant forearm use or in individuals engaged in repetitive forearm activities in daily life. Chronic dislocation can lead to neuritis or neuropathy, particularly in occupations involving frequent forearm use, such as manual workers^3^. Some case reports have shown that patients often experience symptoms of pain and snapping around the medial epicondyle, which can worsen with movement^4^. These symptoms can sometimes be challenging to differentiate from the more common pathologies, such as medial epicondylitis and ulnar nerve entrapment. Our patient is a typical example of such a misdiagnosis. Initial treatment for mild symptoms involves conservative management with nonsteroidal anti-inflammatory drugs (NSAIDs) and physical therapy. Patients with persistent or clear signs of ulnar nerve neuropathy that are unresponsive to these treatments should be considered for surgery to prevent further nerve damage^3^. This case report has been reported in line with the SCARE 2023^5^.

## Case report

**Figure 1:**
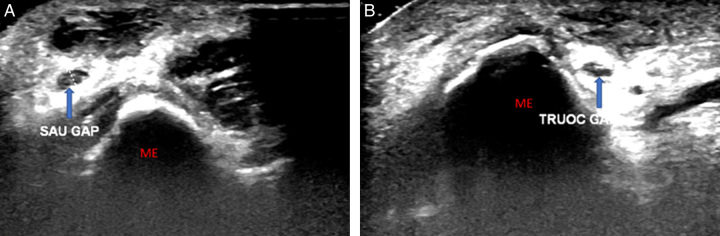
Dynamic ultrasound evaluating the ulnar nerve’s position, (A) over than 90° elbow flexion, (B) elbow extension, (blue arrow) ulnar nerve. ME, medial epicondyle.

**Figure 2:**
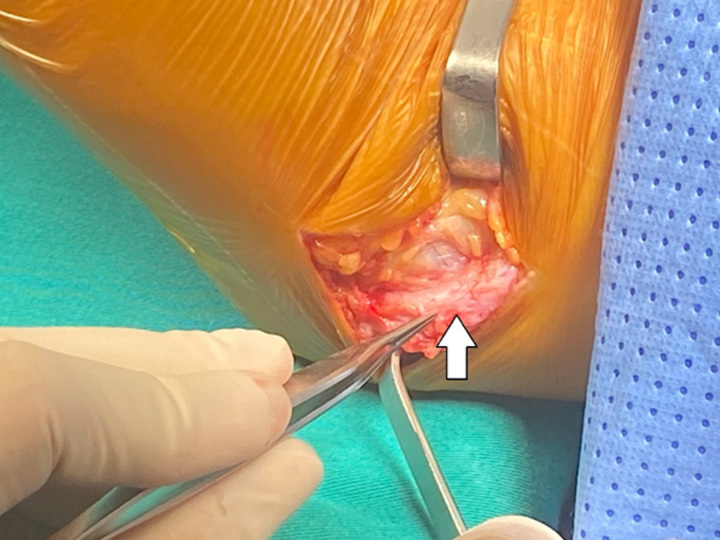
The ulnar nerve dislocates anteriorly around the medial epicondyle upon flexion (white arrow).

The patient is a 51-year-old male with a history of a right elbow injury, which he sustained at a tennis game over 1 year ago due to improper technique. The pain gradually decreased when the patient abstained from sports for a period of time. However, as he returned to sport, the pain progressively increased, and since then, it has become persistent and dull even at rest. The patient visited several local hospitals with a diagnosis of medial epicondylitis and received internal medicine treatment, including NSAIDs, rest, and cold compressions, with the most recent treatment being an intra-articular corticosteroid injection two months ago. Following the treatment, his overall symptoms improved, but the pain still persisted with elbow movement. Magnetic resonance imaging suggested swelling and oedema of the ulnar nerve segment in the cubital region and surrounding soft tissues. Upon examination, we palpated the ulnar nerve and noticed that it would anteriorly dislocate when the elbow was flexed beyond 90° and would return to its original position within the retrocondylar groove upon extension. Tinel’s test was positive, and no complications of nerve injury were observed. Dynamic ultrasound examination confirmed the dislocation of the ulnar nerve from the retrocondylar groove anteriorly, corresponding to elbow flexion and extension beyond 90° (Fig. 1). Consequently, the patient underwent ulnar nerve transposition. During the surgery, after dissecting the ulnar nerve, we found a torn cubital tunnel retinaculum, and the ulnar nerve segment just behind the medial condyle was not covered by the cubital tunnel retinaculum. Intraoperative examination confirmed the nerve’s posterior to anterior movement, corresponding to flexion and extension (Fig. 2).

**Figure 3:**
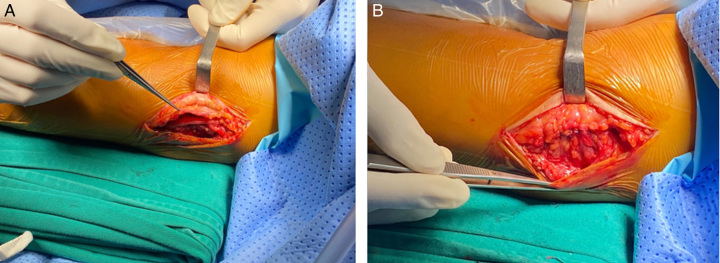
Transposing the ulnar nerve anteriorly, (A) mobilize the ulnar nerve circumferentially, (B) mattress suture technique.

We performed the ulnar nerve anterior transposition surgery using the subcutaneous transposition technique (Fig. 3).

After surgery, the patient’s right elbow was placed in a sling to stabilize the surgical site. After suture removal, the patient could perform daily activities comfortably but was advised to avoid strenuous movements for the next 6 weeks. The patient’s symptoms improved significantly: there was no more pain upon elbow flexion and extension, and signs of neuritis were resolved.

## Discussion

The ulnar nerve originates from the C8 and T1 spinal roots, combining to form the medial cord of the brachial plexus. The ulnar nerve proceeds down the medial aspect of the arm, passing through the intermuscular septum of the triceps and entering the cubital tunnel between the medial epicondyle and the olecranon. The roof of the cubital tunnel is formed by the cubital tunnel retinaculum (Osbourne’s ligament). In some individuals, this roof may be replaced by the anconeus epitrochlearis muscle, which is believed to contribute to ulnar nerve compression. The floor of the tunnel is formed by the medial collateral ligament and elbow joints capsule while the medial epicondyle and olecranon act as walls on either side^6^. This structure serves to protect and stabilize the ulnar nerve at the elbow, preventing the nerve from sliding during flexion and extension.

Studies on the morphology and movement of the ulnar nerve in the cubital tunnel using ultrasound by Okamoto and colleagues have shown that during flexion of the elbow, the ulnar nerve tends to move forward and inward and occasionally passes over the peak of the medial epicondyle, causing subluxation in some patients^7^. Excessive movement can lead to nerve irritation and result in ulnar neuropathy. Yang and colleagues found significant displacement of the ulnar nerve at the cubital region in patients with ulnar neuropathy compared to normal individuals when measuring the distance of ulnar nerve movement to fixed anatomical landmarks using ultrasound^8^.

Therefore, the ulnar nerve can naturally be subluxed or dislocated if Osborne’s ligament is loose or when there are anatomical variations in the medial epicondyle^2^. Additionally, it can also occur due to a tear in Osborne’s ligament following an injury. Several authors have reported postoperative ulnar nerve instability after the removal of Osborne’s ligament for ulnar nerve decompression and agree that the instability of the ulnar nerve is the cause of discomfort and an increased risk of revision surgery after nerve decompression^9–12^. In the case of our patient, we found that the cause was an elbow injury, leading to a torn cubital tunnel retinaculum. The patient did not receive proper treatment, only refraining from sports activities while continuing normal daily activities, which resulted in the healing failure of the retinaculum and the recurrent dislocation of the ulnar nerve. On the other hand, Gangadharan *et al*.^13^ have also reported a case of post-traumatic ulnar nerve subluxation.

Several symptoms and clinical stages of ulnar nerve subluxation were reported by Blattmann in 1851^14^. With the current understanding of the pathology and kinetics of the ulnar nerve, we can now describe the symptoms associated with ulnar nerve instability, which manifest as friction neuritis. Additionally, symptoms are intensified by elbow flexion^3^. However, these symptoms do not always accompany ulnar nerve instability. Endo *et al*.^14^ conducted a study examining 153 healthy individuals, of whom 78 cases showed signs of ulnar nerve instability on dynamic ultrasound. Nevertheless, the authors observed a significant increase in the cross-sectional area of the ulnar nerve, which is considered a risk factor for future ulnar neuropathy. Our patient presented with persistent pain at the medial epicondyle, which persisted even at rest. The pain intensified during flexion and was accompanied by noticeable “snapping” sensations. However, local hospital physicians primarily attributed the symptoms to medial epicondylitis, leading to initial treatment focused on that condition. It is important to recognize that these symptoms can occasionally coexist with ulnar neuritis. The relatively high prevalence of this condition makes it easier for physicians to overlook signs of ulnar nerve inflammation.

When ulnar nerve subluxation is not accurately diagnosed and further progresses, sensory symptoms will manifest at the elbow. Additionally, the ineffectiveness of the medial epicondylitis treatment plan will change the diagnosis to early-stage ulnar nerve entrapment and subsequent treatment. However, lack of progress in this treatment approach and absence of ulnar nerve compression signs on electromyography create confusion and challenges in determining the condition and its appropriate treatment.

Even when ulnar nerve subluxation is correctly diagnosed, physicians occasionally may overlook another rare condition that also causes snapping of the elbow, which is dislocation of the medial portion of the triceps. Therefore, a thorough clinical and subclinical evaluation is necessary to avoid overlooking these rare conditions.

**Figure 4:**
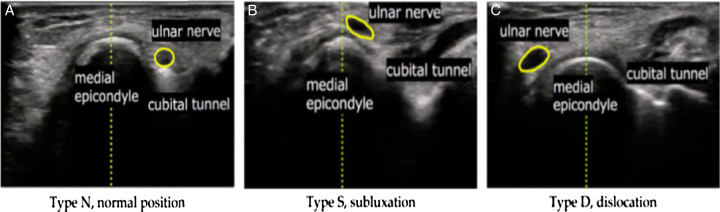
Short-axis images at the cubital tunnel using ultrasonography. (A) Type N, normal position (ulnar nerve was into the cubital tunnel); (B) Type S, subluxation (ulnar nerve was on the tip of the medial epicondyle); and (C) Type D, dislocation (ulnar nerve exceed and positioned anteriorly to tip of the medial condyle)^7^.

Several noteworthy clinical signs of ulnar nerve instability are medial elbow pain, palpable sliding forward and popping out of the ulnar nerve during elbow flexion and returning to its normal position upon elbow extension, ulnar nerve dysfunction manifested at the hand such as Froment’s sign and Tinel sign of the cubital tunnel^3^. Subclinical evaluations can be performed to assess the ulnar nerve function, such as measuring conduction velocity through electroneuromyography, ultrasound assessment of the cross-sectional area of the nerve, or assessment of nerve tension through shear-wave elastography. Magnetic resonance imaging can also be used to observe the morphology and abnormalities of the surrounding soft tissues of the cubital nerve. However, these evaluations only indicate abnormalities in the function and morphology of the ulnar nerve but cannot determine the exact cause and are difficult to identify in patients with asymptomatic ulnar nerve dislocation. Another effective and simple method, that can be performed even in local hospitals, is dynamic ultrasound of the ulnar nerve in two positions: extension and over than 90° flexion^2,3,13^. The classification of ulnar nerve dislocation is also based on the mobile nerve position during elbow flexion, as described by Okamoto *et al*.^7^ (as seen in Fig. 4).

For initial treatment of recurrent ulnar nerve dislocation with mild symptoms, non-surgical treatment with NSAIDs, rest, and refraining from repetitive flexion and extension movements are recommended. Pain symptoms typically diminish, but the snapping sensation may persist. If patients have persistent or severe pain or the ulnar nerve dysfunction symptoms are unresponsive to non-surgical treatment, surgical repositioning of the nerve to the anterior side can be considered^3^. Two main techniques for nerve transpositioning surgery are subcutaneous and submuscular. Although no definitive evidence proves the superiority of one technique over the other, we opted for the subcutaneous technique for this patient due to its simplicity and safety, while avoiding potential complications associated with the submuscular approach such as haematoma, cutaneous nerve branch injury, and joint stiffness^2,3^. Postoperative outcomes are typically favourable, with patients returning to normal activities within 3 weeks and experiencing pain relief after several months of functional recovery^4^.

## Conclusion

Post-traumatic ulnar nerve dislocation is a rare condition, and the recurrent characteristic of it leads to neuritis or neuropathy. The condition can be overlooked or misdiagnosed as medial epicondylitis or early-stage ulnar nerve entrapment. Common subclinical assessments are inadequate to accurately determine this condition. Therefore, a comprehensive clinical examination and appropriate subclinical assessments are necessary for accurate diagnosis. Nerve transposition surgery, which is indicated for cases of persistent pain and unresponsive to non-surgical treatment, provides significant positive outcomes.

## Ethical approval

Ethical approval is exempt/waived at our institution.

## Consent

Written informed consent was obtained from the patient for publication of this case report and accompanying images. A copy of the written consent is available for review by the Editor-in-Chief of this journal on request. The patient received an explanation of the procedures and possible risks of the surgery, and gave written informed consent.

## Source of funding

We declare no funding for this research.

## Author contribution

D.T.T. contributed to perform the operation, revising, and approval for publishing. M.N.H., Q.T. contributed to assist the operation, data collection, analysis and interpretation, manuscript drafting. V.V.D. contributed to data collection, analysis and interpretation, manuscript drafting.

## Conflicts of interest disclosure

We declare that we have no known competing financial interests or personal relationships with anyone that could have appeared to influence the work reported in this paper.

## Research registration unique identifying number (UIN)

Our article is a case report. It is not human study.

## Guarantor

D.T.T. is the guarantor of this manuscript.

## Provenance and peer review

Not commissioned, externally peer-reviewed.
